# Development, Characterization, and *In Vitro* Biological Performance of Fluconazole-Loaded Microemulsions for the Topical Treatment of Cutaneous Leishmaniasis

**DOI:** 10.1155/2015/396894

**Published:** 2015-01-12

**Authors:** Marcela Brito Oliveira, Giovana Calixto, Márcia Graminha, Hugo Cerecetto, Mercedes González, Marlus Chorilli

**Affiliations:** ^1^Department of Drugs and Medicines, School of Pharmaceutical Sciences, UNESP, Rodovia Araraquara-Jaú, km. 1, Campus, 14801-902 Araraquara, SP, Brazil; ^2^Department of Clinical Analysis, School of Pharmaceutical Sciences, UNESP, Rodovia Araraquara-Jaú, km. 1, Campus, 14801-902 Araraquara, SP, Brazil; ^3^Departamento de Química Orgánica, Facultad de Química-Facultad de Ciencias, Universidad de la República, 11400 Montevideo, Uruguay

## Abstract

Cutaneous leishmaniasis (CL) is a resistant form of leishmaniasis that is caused by a parasite belonging to the genus* Leishmania*. FLU-loaded microemulsions (MEs) were developed by phase diagram for topical administration of fluconazole (FLU) as prominent alternative to combat CL. Three MEs called F1, F2, and F3 (F1—60% 50 M phosphate buffer at pH 7.4 (PB) as aqueous phase, 10% cholesterol (CHO) as oil phase, and 30% soy phosphatidylcholine/oil polyoxyl-60 hydrogenated castor oil/sodium oleate (3/8/6) (S) as surfactant; F2—50% PB, 10% CHO, and 40% S; F3—40% PB, 10% CHO, and 50 % S) were characterized by droplet size analysis, zeta potential analysis, X-ray diffraction, continuous flow, texture profile analysis, and* in vitro* bioadhesion. MEs presented pseudoplastic flow and thixotropy was dependent on surfactant concentration. Droplet size was not affected by FLU. FLU-loaded MEs improved the FLU safety profile that was evaluated using red cell haemolysis and *in vitro* cytotoxicity assays with J-774 mouse macrophages. FLU-unloaded MEs did not exhibit leishmanicidal activity that was performed using MTT colourimetric assays; however, FLU-loaded MEs exhibited activity. Therefore, these MEs have potential to modulate FLU action, being a promising platform for drug delivery systems to treat CL.

## 1. Introduction

Leishmaniasis is an anthropozoonosis with natural foci that affects 12 million people worldwide, with approximately 1-2 million new cases occurring every year, and persists in tropical and subtropical regions [1].

It is caused by a parasite belonging to the genus* Leishmania*, in which infection is spread in wild ecotopes by the bite of the infected female sandfly (Phlebotomine) in their vertebrate hosts [2–4].

These parasites can originate two types of diseases: visceral or cutaneous leishmaniases. Although the visceral form is more aggressive, the cutaneous leishmaniases (CL) have received considerable attention in recent research because most patients are resistant to current treatments [5].

The first-line chemotherapy to combat CL for over 60 years is the pentavalent antimonials, which are Sb-(V) compounds. Amphotericin B, pentamidine isethionate, miltefosine, and paromomycin are also available as alternatives therapies, but their use is also limited because of toxicity or high treatment costs [6].

Hence, the searching for alternatives to conventional treatment for CL has been shown to be extremely valuable. One of these alternatives is administration of imidazole antifungals such as fluconazole (FLU), also known as [2-(2,4-difluorophenyl)-1,3-bis (1H-1,2,4-triazol-1-yl) propan-2-ol], that presents moderate lipophilicity (log 0.5), renal excretion of over 80% of the unchanged drug, a half-life of 30 hours, 12% binding plasma protein, and over 90% bioavailability when orally administered [7–11].

FLU can favor the topical treatment of CL, because this drug presents high affinity for keratin prolonging its retention in the skin [12]; moreover FLU acts by inhibiting the P450 cytochrome that blocks the enzyme 14-*α*-demethylase, which is involved in the synthesis of the parasitic ergosterol membrane, thus helping to heal localised lesions [13–17].

The clinical efficacy of FLU against CL was assessed by a randomized, double-blind, placebo controlled trial. For this, a dose of 200 mg daily was administrated for six weeks and the results demonstrated that FLU can be safe and effective to treat the CL [18].

Another study evaluated the efficacy of a cream containing 1% FLU. The results showed that none of the animals was cured by cream [19]. This limitation can be ascribed to the low skin penetration of FLU, because even though studies show that FLU has high affinity for keratin, further studies have reported that high concentration of FLU in the skin is due to infinite dose of FLU, which is not reproduced in the clinical treatment [12]. Therefore, it becomes necessary to develop novel topical FLU delivery systems to make viable the clinical treatment.

An interesting and unexplored alternative to overcome these obstacles is to design nanostructured drug delivery systems incorporated with FLU. A lot of attention has been given to micro- and nanostructured surfactants systems, as microemulsions (ME), which are clear, isotropic, and thermodynamically stable colloidal systems obtained spontaneously by surfactant—cosurfactant combinations [20–23].

ME systems present features, such as (i) controlling drug release; (ii) protecting drug against thermal degradation; (iii) protecting drug against photo bleaching; (iv) reducing the drug dose; (v) minimizing the potential of side effects [24–28], which make them stand out as a potential topic drug delivery system for CL treatment seeing that the conventional dosage forms such as gels, creams, and ointments against CL have not shown an effective action against all of the* Leishmania* species [16]; moreover such formulations presented toxic and irritating, as well as ardency and burning, sensations restricting its use [29].

The other property which makes ME a drug delivery system far superior to conventional systems is its ability to modify the diffusional barrier of the skin, improving the partitioning of FLU into the skin [30]; thereby this may increase the FLU leishmanicidal activity.

Therefore, the aim of this study was to evaluate the potential of a FLU-loaded ME for the treatment of CL. The formulations were structurally characterized using droplet size analysis, zeta potential analysis, X-ray diffraction, continuous flow, texture profile analysis, and* in vitro* bioadhesion assays. The safety profile was evaluated using red cell haemolysis and* in vitro* cytotoxicity assays and the* in vitro* leishmanicidal activity was evaluated using a MTT colorimetric assay.

## 2. Materials and Methods

### 2.1. Materials

Fluconazole was purchased from DEG (Sao Paulo, Sao Paulo, Brazil). Sodium oleate and cholesterol were purchased from Sigma-Aldrich (St Louis, Missouri, USA). Soy phosphatidylcholine was purchased from Lucas Meyer Gmbh & Co (Hamburg, Hamburg, Germany). Phosphate buffer solution was prepared from sodium monohydrogen phosphate and dihydrogen phosphate that were purchased from Merck (Darmstadt, Germany). Polyoxyl-60 hydrogenated castor oil was purchased from Pharma Special (Itapevi, Sao Paulo, Brazil). Pentamidine isethionate was purchased from Sigma-Aldrich (St Louis, MO, USA).

### 2.2. Preparation of Formulations

It was constructed as a pseudoternary phase diagram using a transparent bottle that was filled with adequate amounts of cholesterol (oil phase), 50 M of phosphate buffer at pH 7.4 (aqueous phase), and a mixture of surfactants, soy phosphatidylcholine, polyoxyl-60 hydrogenated castor oil, and sodium oleate, in a proportion of 3 : 8 : 6. The mixture was sonicated using Sonic Boom (Sonics, Vibra-Cell, Newtown, Connecticut, USA), which was rated at 220 watts and operated in a discontinuous manner for 20 minutes at intervals of 30 seconds to 1 minute: an ice bath was used throughout the sonication process [31–33].

The formulations were classified visually as follows: transparent viscous systems (TVS), viscous and opaque systems (VOS), opaque liquid systems (OLS), liquid and transparent systems (LTS), and phase separated systems (PS). 1% FLU (w/w) was incorporated by dissolving the drug powder directly in the studied formulations.

### 2.3. Physicochemical and Structural Characterisation of Systems

#### 2.3.1. Droplet Size Analysis (Dynamic Light Scattering)

The droplet size analysis was determined using dynamic light scattering with a ZetaPlus (Brookhaven Instrument Corporation, Holtsville, NY, USA). The samples were diluted and placed in the analysis chamber such that the laser beam passed through the entire length of the dispersions. The system temperature was maintained at 20°C, the laser wavelength was 532 nm, and the refractive index was consistent with the rate observed for each sample. Ten measurements were made of the diameter and polydispersity index (PDI) of the droplets for each sample in triplicate, and the total duration of the experiment was 5 min [34].

#### 2.3.2. Zeta Potential Analysis

The zeta potential (*ζ*mV) was also determined using a dynamic light scattering with a ZetaPlus (Brookhaven Instrument Corporation, Holtsville, NY, USA), where the droplets of the formulation were subjected to a fixed voltage, and the *ζ*mV was calculated using the values provided by the apparatus. Ten measurements of the electrophoretic mobility were made for each sample (*n* = 3) [35].

#### 2.3.3. X-Ray Diffraction

An X-ray diffractometer Siemens D5000 (Siemens, Munich, Bavaria, Germany) with copper radiation monochromatizated by graphite crystal was used to determine the degree of crystallinity or the amorphous state of the samples, the ME components (sodium oleate, soybean phosphatidylcholine, castor oil, polyoxyl-60-hydrogenated, and cholesterol), and FLU. The scanning speed was 0.1 seconds at each 0.050 to 2*θ*° ranging from 4° to 70° [35].

#### 2.3.4. Texture Properties Analysis

Texture profile analyses of the samples were performed using a TA-XTplus texture analyzer (Stable Micro Systems, Surrey, UK) in TPA mode. Ten-gram samples were placed in 50 mL Falcon tubes and centrifuged at 4000 rpm for 10 minutes (Eppendorf 5810R, New York, USA). The samples were placed 10 mm above the analytical probe, which reached a constant speed of 1 mm s^−1^ by detecting the triggering strength for 2 min, after which the probe was descended 10 millimetres into the sample. The probe was then returned to the surface at a speed of 0.5 mm s^−1^, and a second compression was initiated after 5 seconds. The resulting force-time curve was used to calculate various mechanical parameters, such as the hardness, the compressibility, the adhesion, and the cohesiveness. The assays were performed in triplicate at 25°C [36, 37].

#### 2.3.5. Bioadhesion Assay

The bioadhesive force between the pig ears' skin and the samples was assessed by detachment test using a TA-XTplus texture analyzer (Stable Micro Systems, Surrey, UK). The porcine ear skin was obtained from a local slaughterhouse and cleaned. The injured skins were discarded. The skin was removed from the cartilage with a scalpel and a 400 *μ*m thick layer of the stratum corneum and epidermis was separated from the adipose tissue with a dermatome (Nouvag TCM 300, Goldach, USA). These prepared skins were frozen for the maximum time of four weeks. On the day of the experiment, the skin was thawed in physiological saline solution, containing 0.9% (w/v) NaCl (Merck), at 25 ± 0.5°C for 30 min; then, its hairs were cut with scissors and it was attached to the lower end of a cylindrical probe (diameter 10 mm) with a rubber ring.

The samples were packed into shallow cylindrical vessels and the test started lowering the analytical probe, which contained the skin at a constant speed (1 mm·s^−1^) onto the surface of the sample. The skin and the sample were kept in contact during 60 s and no force was applied during this interval. After 60 s, the skin was drawn upwards (0.5 mm·s^−1^) until the contact between the surfaces was broken. The bioadhesive force of the samples was measured in the maximum detachment force as the resistance to the withdrawal of the probe, what reflects the bioadhesion characteristic. Seven replicates were analysed at 32 ± 0.5°C [36, 37].

#### 2.3.6. Rheological Study

Continuous flow was analysed on a controlled-stress AR2000 (TA Instruments, New Castle, DE, USA) equipped with parallel plate geometry (40 mm diameter) and sample gap of 200 *μ*m, at 32 ± 0.1°C, in triplicate. Samples of the hydrogel were carefully applied to the lower plate, ensuring that sample shearing was minimized, and allowed to equilibrate for 3 min prior to analysis.

Continual testing was performed using a controlled shear rate procedure in the range from 0.01 to 100 s^−1^ and back, each stage lasting 120 s, with an interval of 10 s between the curves. The consistency index and flow index were determined from the power law described in (1) for a quantitative analysis of flow behavior [34]:

(1) $$\begin{array}{c} \tau =k\cdot \gamma^{\eta }, \end{array}$$

where “*τ*” is shear stress; “*γ*” is shear rate; “*k*” is consistency index; and “*η*” is flow index [36, 37].

### 2.4. *In Vitro* Biological Assays

#### 2.4.1. Erythrocyte Haemolysis

Before use, freshly collected human blood (O positive) was washed three times with 0.01 M Tris-HCl at pH 7.4, which contained 0.15 M NaCl (Tris-saline). A suspension of 1% (v/v) erythrocytes consisting of packed red blood cells resuspended in Tris-saline was prepared. A FLU-loaded ME, a FLU-unloaded ME, and a FLU-free sample were dissolved in Tris-saline to a final concentration of 27 *μ*M. A 1% (v/v) Triton X-100 solution was used as a positive control (which corresponded to 100% lysis). After incubation for 1 h at 37°C, the samples were centrifuged at 3000 ×g for 2 min. Aliquots of 100 *μ*L of the supernatant were transferred to 96-well microplates, and the absorbance was determined at 405 nm using a BioRad Model 3550-UV (USA) microplate reader. The assay was performed in triplicate. The percentage of haemolysis was calculated using the following equation: % haemolysis = (test sample absorbance/absorbance at 100% lysis) × 100 [38, 39].

#### 2.4.2. “*In Vitro*” Unspecific Cytotoxicity

The mammal cytotoxicity of the formulations was studied* in vitro* using J-774 macrophages (ECACC 85011428) derived from a tumor of a female BALB/c mouse as cellular models. Cells were seeded at a density of 2.5–10.0 × 10^5^ cells/well in 96-well plates in flat-bottomed microplates (Nunclon) and exposed to different doses of the formulations and FLU-free samples (at concentrations of 18.6, 10, 5, and 1 *μ*M) or to vehicle control for 48 h. After treatment, the compounds were removed, and the cells were washed once with PBS. The cell viability was then colourimetrically assessed by measuring the mitochondrial-dependent reduction of MTT to formazan. For this purpose, the cells and MTT (0.4 mg/mL) were incubated in air at 37°C for 3 h. After the incubation period, the supernatant was removed, and the formazan crystals were dissolved using 180 *μ*L of DMSO. The plates were shaken for 10 min, and the optical densities were measured at 560 nm using a multiwall spectrophotometer. Each concentration was assayed three times, and six additional controls (consisting of the cells in medium) were used in each test. The cells were inactivated for at least 2 hours in a solution of hypochlorite (10,000 ppm) prior to disposal to drain with an excess of water. The data were presented in terms of the IC_50_ values, that is, the compound concentration required to reduce the cells by half. The cell viability was calculated using the following equation: cell viability (%) = [OD_560_ (sample)/OD_560_^4^] × 100 [38].

#### 2.4.3. Evaluation of Leishmanicidal Activity

Promastigotes of* L. amazonensis* MPRO/BR/1972/M1841-LV-79 strain were maintained at 28°C in liver-infusion tryptose supplemented with 10% foetal calf serum (FCS). Cultured promastigotes at the end of the exponential growth phase were seeded at 8 × 10^6^ parasites/mL in 96-well flat-bottomed plates (Costar). The F1, F2, F3, F1D, F2D, and F3D formulations and FLU were added to the parasite suspension to obtain final concentrations between 1.6 *μ*g/mL and 100 *μ*g/mL, which were then incubated at 28°C for 72 h. The assays were carried out in triplicate. A stock solution of pentamidine isethionate at a concentration of 16.7 mg·mL^−1^ was prepared in sterile, deionised water to serve as a reference drug. The leishmanicidal effects of the samples were assessed using a modified 3-[4,5-dimethylthiazol-2-yl]-2,5-diphenyltetrasodium bromide (MTT) method [35]. The absorbances of the samples were measured at 490 nm [40]. The concentration corresponding to 30% of parasite growth inhibition was expressed as the inhibitory concentration (IC_30_).

### 2.5. Statistical Analyses

The data were analysed to obtain the mean and standard deviation and compared using the analysis of variance (ANOVA). In the data comparison, a one-way Tukey test was used to assess significant differences between the samples, where values with *P* < 0.05 were considered to be statistically significant. The program Origin 7.0 SRO was used in the data treatment.

## 3. Results and Discussion

### 3.1. Preparation of Formulations

**Figure 1:**
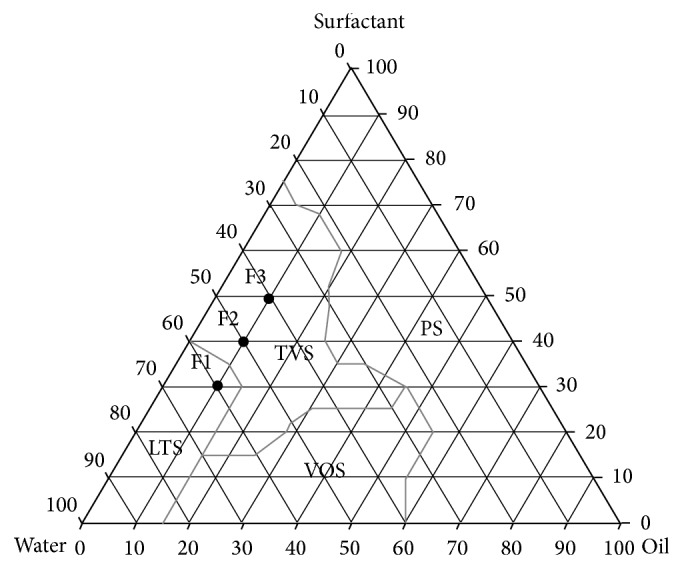
Ternary phase diagram. The marked areas represent the following. PS: phase separation; VOS: viscous opaque system; TVS: transparent viscous system; and LTS: liquid transparent system. The points designated F1, F2, and F3 are the studied MEs.

Figure 1 shows the phase diagram for the system with the characteristic LTS (liquid transparent systems), TVS (transparent viscous systems), PS (phase separation), and VOS (viscous opaque systems) regimes. LTS regime was observed at low oil concentrations (10%) and low surfactant concentrations (10–30%); TVS regime was observed for surfactant concentrations between 20 and 65% and oil concentrations of 10 to 40%; VOS regime was found in the lower central region of the diagram at low oil concentrations of 20–50% and surfactant concentrations of 10–20%; and the PS regime was found at low water concentrations (10–30%) and was independent of the surfactant concentration in the formulations.

There were 3 selected formulations of the diagram system, and the rates of the components are showed in Table 1. The oil phase was fixed because the cholesterol interacts with the biological membrane that can lead to increased membrane permeability, aggregation process, enzymatic activity, cell fusion, and the stiffness, size, and shape of this membrane. Furthermore, the cholesterol molecule exhibits both polar and nonpolar characteristics and can guide the hydroxyl ion into the aqueous phase and the carbon chain towards the surfactant [41–44].

### 3.2. Physicochemical and Structural Characterisation of Systems

#### 3.2.1. Droplet Size Analysis (Dynamic Light Scattering)

Table 2 shows the particle sizes obtained from analyzing the formulations: F1—180.500 to 181.200 nm, F2—618.933 to 928.833 nm, and F3—317.800 to 357.633 nm. The addition of FLU only changed the particle size for the F2 sample, which exhibited a significant increase in the droplet diameter following drug addition. This result may be explained by the coalescence of droplets over time because most of the newly formed droplets in the formulation can be found at the surface of the vial to which the drug was added, whereas the droplets with the highest age in the formulation were at the bottom of the storage vessel of the sample. The PDI is an index that reflects the relative homogeneity of particle sizes in a sample. The PDI values varied from 0.174 to 0.703 demonstrating a good size distribution of the oil droplets in the ME system, reflecting the size homogeneity of the droplets in the bulk ME [45, 46].

#### 3.2.2. Zeta Potential Analysis

Table 2 also shows that the zeta potential values for the samples ranged from −38.600 ± 1.967 mV to −55.867 ± 3.044 mV. The negative charge resulted from the microemulsion surfaces being coated upon contacting the liquid because a large repulsion guarantees microemulsion stability. This repulsion arises from the negatively charged components of the formulations and ionic forces [47].

Soy phosphatidylcholine and sodium oleate have an ester group from which the sodium atom can dissociate, and the cholesterol structure has a free hydroxyl group. Drug addition did not change the zeta potential values of any of the tested samples.

#### 3.2.3. X-Ray Diffraction

X-ray diffraction was used to determine whether the materials were crystalline or amorphous in nature. Intense and sharp peaks with narrow halos in the diffraction pattern of a sample are characteristic of crystalline materials; the absence of intense peaks and broad halos are characteristic of amorphous materials [48, 49].

**Figure 2:**
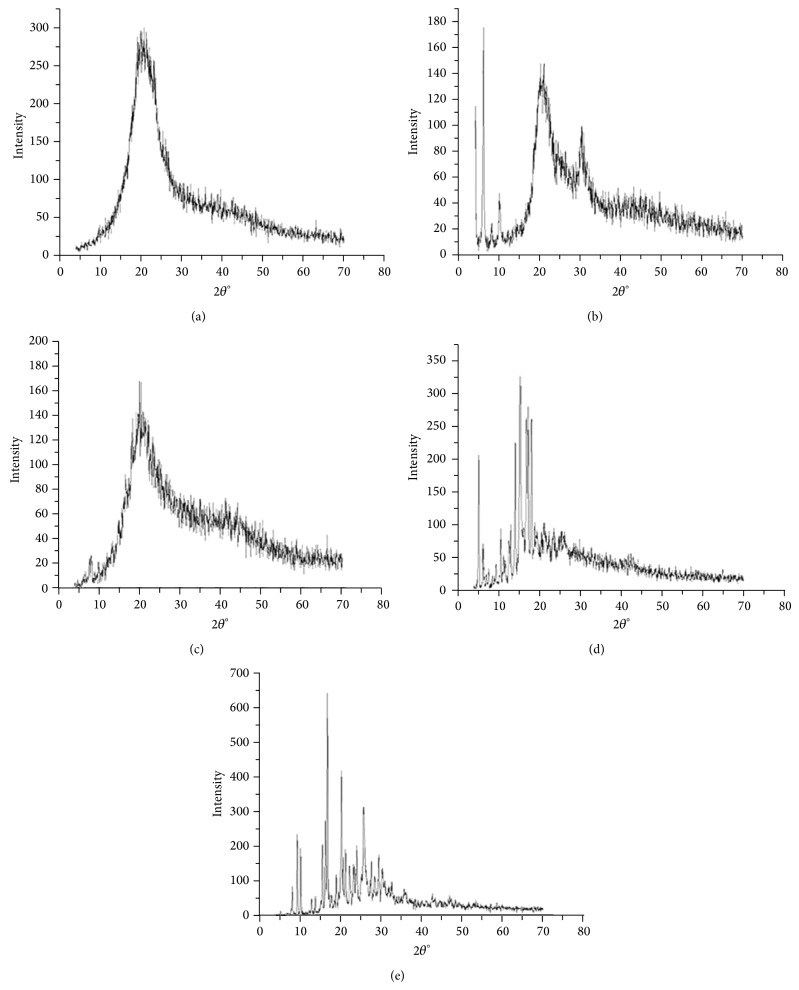
X-ray diffraction spectra for microemulsion components: (a) sodium oleate, (b) cholesterol, (c) polyoxyl-60 hydrogenated castor oil, (d) fluconazole, and (e) soy phosphatidylcholine.

Figure 2 shows the diffractogram obtained for ME components. The intense and defined diffraction peaks for sodium oleate (Figure 2(a)), cholesterol (Figure 2(b)), and fluconazole (Figure 2(d)) were crystalline below 10° (2*θ*), between 5 and 20° (2*θ*), and between 5° and 35° (2*θ*), respectively, that are characteristic of crystalline structures. The diffractograms obtained for polyoxyl-60 hydrogenated castor oil (Figure 2(c)) and soy phosphatidylcholine (Figure 2(e)) exhibited low intensity peaks with broad halos; thus, these components are considered amorphous structures [50].

**Figure 3:**
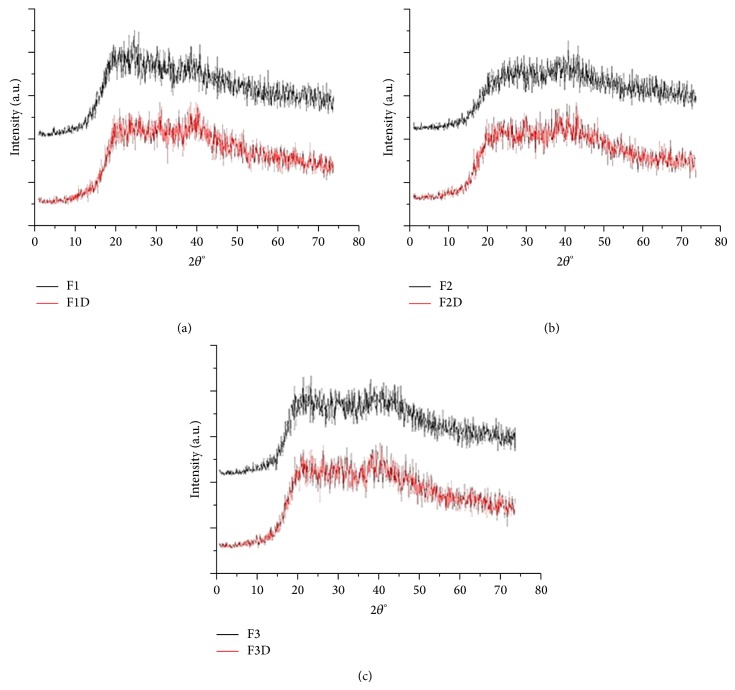
X-ray diffraction spectra of FLU-unloaded MEs (F1, F2, and F3) and FLU-loaded MEs (F1D, F2D, and F3D): (a) F1 and F1D, (b) F2 and F2D, and (c) F3 and F3D.

The absence of high peaks in Figure 3 shows that the FLU-loaded ME and FLU-unloaded ME exhibited low intensity peaks with amorphous halos present that is common and desirable characteristic for microemulsion systems [46].

#### 3.2.4. Texture Properties Analysis

The difference in the mechanical characteristics of the FLU-unloaded and -loaded MEs was shown in Table 3.

Hardness, compressibility, and adhesion decreased only when the drug was added to the F2 sample while F1 and F3 were not influenced by FLU.

The hardness and compressibility are related to the strength of the ME structure under compression and express the applicability of the formulation to the desired site. A low value of these parameters, as obtained for the FLU-loaded MEs, has been reported as an advantage for dermal application of formulations because this parameter significantly affects spreadability of the ME [36, 51].

Adhesiveness is the work required to overcome the attractive forces between the surface of the MEs and the probe and reflects alterations in product viscosity [51]. In our study, the lowest adhesiveness value was obtained with the F1D classified as LTS, as demonstrated above.

Finally, cohesiveness parameter shows the degree of difficulty in breaking down the MEs internal structure and it is calculated through the ratio of the area under the first and second immersion. The high cohesiveness values of the ME indicate the ability to prepare homogeneous formulations [37, 51].

#### 3.2.5. Bioadhesion Assays

**Figure 4:**
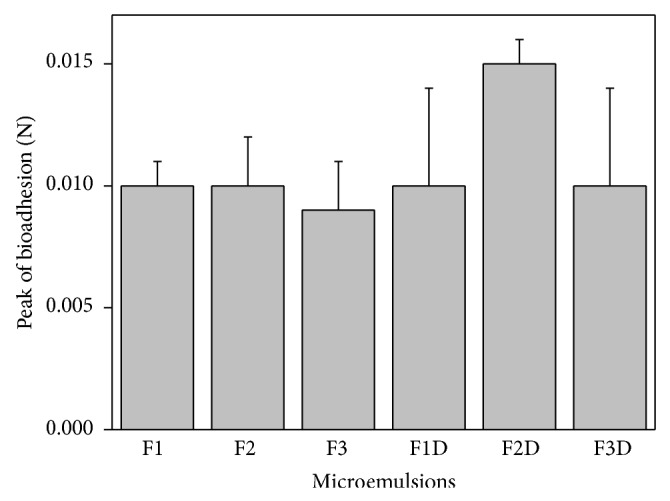
Peak of bioadhesion (N) of FLU-unloaded MEs (F1, F2, and F3) and FLU-loaded MEs (F1D, F2D, and F3D). Each value represents the mean (± standard deviation) of at least seven replicates. Data were collected at 32 ± 0.5°C. No statistically significant difference was detected.

Figure 4 shows the peak force corresponding to the maximum force between the probe and the tissue as a function of time. The data shows that the bioadhesion of all of the samples was unchanged by drug addition neither by the composition of the MEs.

The bioadhesive stability of FLU-loaded ME is an important physicochemical parameter for topical application that allows the interaction of the formulations with superficial epithelial cells resulting in a closest contact with the biological surface by an extended time and also an increased local gradient of drug concentration in the target site [36, 52].

Bachhav and Patravale developed microemulsion based gel for the vaginal delivery of fluconazole that showed significantly higher antifungal activity as compared to that of commercial product. Thus, bioadhesive MEs can improve the performance of the drug [53].

#### 3.2.6. Rheological Study

**Figure 5:**
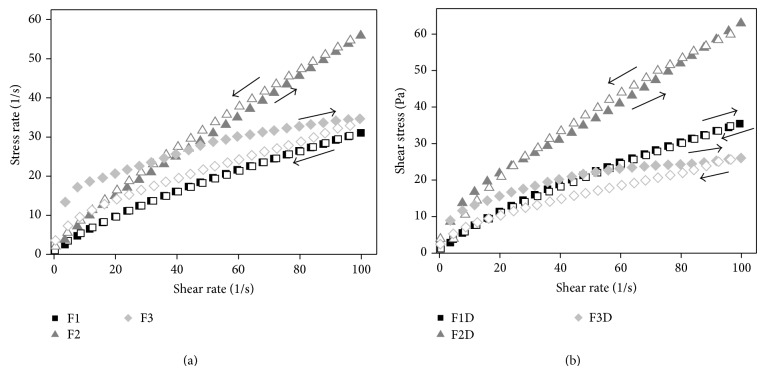
(a) Flow rheograms of FLU-unloaded MEs F1 (**■**), F2 (▲), and F3 (◆). (b) Flow rheograms of FLU-loaded MEs F1D (**■**), F2D (▲), and F3D (◆). Closed symbol represents up curve and open symbol represents down curve. Standard deviations have been omitted for clarity; however, in all cases, the coefficient of variation of triplicate analyses was less than 10%. Data were collected at 32 ± 0.5°C.

The flow curves of FLU-unloaded MEs and FLU-loaded MEs are presented in Figure 5, in which the relation between the shear rate (Pa) and shear stress (1/s) evidenced that all ME's exhibited non-Newtonian “shear thinning” pseudoplastic flow behaviour.

From the data obtained by (1) and shown in Table 4, it was confirmed that all MEs exhibited shear thinning pseudoplastic behavior because all *n*-values obtained were less than a unity (*n* < 1). Furthermore, the presence of FLU did not alter the flow behaviour of the MEs.

This pseudoplastic behavior may be due to breaking of the organized structures that leads to forming less organized structures such as droplets when the stress is applied to the MEs. It is a desirable attribute for skin pharmaceutical products, because the formulation begins to flow easily after the stress application, leading to a good spreading during application and a formation of a uniform film on the skin surface. After withdrawal of the stress, the viscosity of formulation increases instantaneously avoiding its outflow [36]. Moreover, *n*-values decreased with the increase of surfactant concentration, showing a trend of increasing the shear thinning characteristic of the studied systems.

As can be also seen in Table 4, the consistency index (*K*) increased with the increase of surfactant concentration. Thus, the highest surfactant concentration increased the apparent viscosity of the ME that may due to the different formation and more organized microstructure [36].

As a further study, the thixotropy of MEs was investigated. As can be seen in Figure 5, a notable thixotropic response was only observed for FLU-unloaded F3 and FLU-loaded F3, because the descending curve does not overlap with the ascending one.

This phenomenon demonstrated again the crucial role of the surfactant concentration into the ME for its flow behaviour. In this case, the increase of surfactant content led to the formation of new structures when the shear rate was increased. Therefore, a longer period of time to restore the relaxed molecular configuration was necessary, what has provided conditions for the appearance of thixotropy.

For the other studied microemulsions, there was a fast recovery of their microstructure showing to be a time-independent structure with the upward and the downward curves overlapped.

### 3.3. *In Vitro* Biological Assays

The safety profile was evaluated using red cell haemolysis and* in vitro* cytotoxicity assays. Erythrocyte membranes have been studied extensively because erythrocyte cells can be obtained by venous puncture, and the membranes are easily isolated by centrifugation. Thus, erythrocytes are a good model for drug-membrane interactions and can provide information about changes in lipid composition, enzymes, or other membrane proteins [54].

FLU solutions resulted in the lysis of 4.72 ± 0.98% of the erythrocyte membranes. FLU-unloaded MEs (F1, F2, and F3) resulted in the lysis of 1.27 ± 0.52%, 1.42 ± 0.43%, and 1.54 ± 0.47% of the erythrocyte membranes, respectively. FLU-loaded MEs (F1D, F2D, and F3D) resulted in the lysis of 3.23 ± 1.24%, 3.45 ± 0.46%, and 3.54 ± 1.15%, respectively, of the erythrocyte membranes, showing that the ME could decrease the lysis power of FLU. Thus, all of the systems showed tolerable erythrocyte haemolysis. Triton X-100, which is a known haemolytic agent, was used as a positive control in the study and showed 100% haemolysis of erythrocytes, thus validating the experiment.

The overall results of the haemolysis study indicated that treatment with the developed lipidic systems was less toxic than previously developed treatments and could be potentially used for therapeutic applications [39].

**Figure 6:**
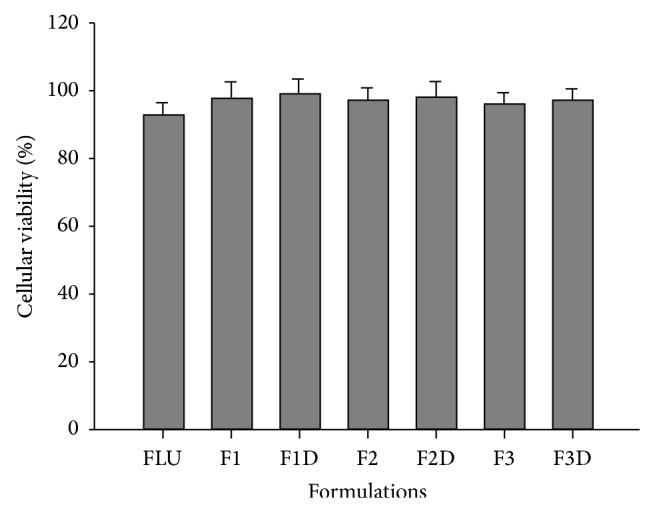
% cellular viability for FLU-free samples, FLU-unloaded MEs (F1, F2, and F3), and FLU-loaded MEs (F1D, F2D, and F3D). No statistically significant difference was detected.

*In vitro* cytotoxicity studies were performed using J-774 mouse macrophages as cellular models. The data are shown as a percentage of the cellular viability in Figure 6.

The cell viability results showed that the FLU-free samples did not kill normal macrophages; that is, the cellular viability was greater than 92%. Moreover, the FLU-unloaded and FLU-loaded MEs also did not show toxic activity.

Therefore, these results showed that these MEs are safety and biocompatibility formulations to eukaryotic cells. Abbasalipourkabir et al. showed cytotoxicity effects from the adherence of particles to cell membranes, particle internalisation, and degradation of products in the cell culture medium or inside the cells. However, the susceptibility of different cell types can be different for different particulate carriers. Systems containing natural lipids should be well-tolerated by living organisms [55, 56].

#### 3.3.1. Evaluation of Leishmanicidal Activity

The parasites that cause leishmaniasis have two evolutionary forms: the amastigote form (which is the form that infects humans by developing inside immune cells, i.e., macrophages) and the promastigote form (the infective vector that develops inside the digestive tract of insects) [57, 58].

Even though the amastigote form causes cutaneous lesions to develop in humans, it is not used in screening studies for new drugs or studies on incorporating the compound into release systems to improve its effectiveness. Instead, the promastigotes of* Leishmania* are used because they are simple to work with, care for, and grow in a short time, which is an advantage in the large-scale screening of potential new drugs [58–61].

The promastigote form is also used in the triage of new drugs to confirm the action of the active principle and as a surrogate for the amastigote (intracellular macrophage) form because both forms exhibit similar characteristics in metabolic pathways [60].

Antileishmanial assays were performed using FLU, FLU-unloaded ME (F1, F2, and F3), and FLU-loaded ME (F1D, F2D, and F3D) against the promastigote forms of* L*.* amazonensis* and the IC_30_ values are shown in Table 5.

There was a reduction in the parasite promastigote forms for FLU-loaded ME, which may suggest that the drug action was potentiated when incorporated into the microemulsions (ME).

The most promising result was obtained when the F3D formulation consists of 40% aqueous phase, 50% surfactant, and 10% oil phase. So, this result may suggest that the highest concentration of the surfactant can improve the fluconazole solubilisation in this microemulsion; besides the drug contact with the parasite membrane can increase by reducing the surface tension [62].

Furthermore, the drug penetration into skin is easier when the formulation is thixotropic, as F3D formulation, since the thixotropic characteristic plays a crucial role in the therapeutic efficacy of the pharmaceutical formulations by improving retention time at the administered site besides enhancing the drug systemic bioavailability [63, 64].

Therefore, the components concentration-defined microemulsion and thixotropic formulations can potentiate the antileishmanial treatment.

## 4. Conclusion

This study demonstrated that it is possible to obtain amorphous microemulsion prepared with cholesterol as oil phase, 50 M phosphate buffer at pH 7.4 as aqueous phase, a mixture of soy phosphatidylcholine, polyoxyl-60 hydrogenated castor oil, and sodium oleate in a proportion of 3 : 8 : 6 as surfactant. The set of results showed that all microemulsions presented a pseudoplastic flow and thixotropy dependent on surfactant concentration. Droplet size was not affected by fluconazole drug. FLU-loaded MEs showed antileishmanial activity in the* in vitro* biological assays, being that ME with the highest concentration of surfactant in its composition and greater thixotropy demonstrated the major antileishmanial activity. Therefore, these MEs should be further investigated since they exhibited promising properties for using as platform for drug delivery systems to treat skin diseases such as cutaneous leishmaniasis.

## Figures and Tables

**Table 1:** Rates of the components of FLU-unloaded MEs (F1, F2, and F3) and FLU-loaded MEs (F1D, F2D, and F3D). Oil phase is cholesterol; aqueous phase is 50 M of phosphate buffer at pH 7.4; surfactant is a mixture of soy phosphatidylcholine, polyoxyl-60 hydrogenated castor oil, and sodium oleate in a proportion of 3 : 8 : 6. LTS: liquid transparent system. VTS: viscous transparent system.

	Formulations (% w/w)					
	F1	F2	F3	F1D	F2D	F3D
Aqueous phase	60	50	40	60	50	40
Oil phase	10	10	10	10	10	10
Surfactant	30	40	50	30	40	50
Fluconazole	—	—	—	1	1	1
O : S ratio	0,33	0,25	0,20	0,33	0,25	0,20
Phases' classification	LTS	VTS	VTS	LTS	VTS	VTS

**Table 2:** Droplet size, polydispersion index, and zeta potential of FLU-unloaded MEs (F1, F2, and F3) and FLU-loaded MEs (F1D, F2D, and F3D). Each value represents the mean (± standard deviation) of three replicates.

Formulations	Droplet size (nm)	PDI	Zeta potential (mV)
F1	180.500 ± 0.721	0.174 ± 0.013	−40.000 ± 1.758
F1D	181.200 ± 1.609	0.228 ± 0.012	−38.600 ± 1.967
F2	618.933 ± 28.612	0.615 ± 0.123	−43.000 ± 0.608
F2D	928.833 ± 34.186	0.703 ± 0.079	−45.167 ± 2.316
F3	317.800 ± 9.364	0.686 ± 0.132	−54.567 ± 2.608
F3D	357.633 ± 18.240	0.568 ± 0.083	−55.867 ± 3.044

**Table 3:** Compressibility, hardness, adhesion, and cohesion of FLU-unloaded MEs (F1, F2, and F3) and FLU-loaded MEs (F1D, F2D, and F3D). Each value represents the mean (± standard deviation) of three replicates at 32°C.

Formulations	Mechanical parameters			
	Hardness (g)	Compressibility (g·s)	Adhesion (g·s)	Cohesion
F1	0.327 ± 0.019	27.139 ± 0.502	1.085 ± 0.596	0.851 ± 0.023
F1D	0.189 ± 0.065	26.935 ± 1.942	0.854 ± 0.089	0.893 ± 0.041
F2	3.620 ± 0.198	53.784 ± 7.923	29.12 ± 6.44	0.728 ± 0.034
F2D	1.249 ± 0.164^*^	33.904 ± 5.133^*^	17.59 ± 5.071^*^	0.840 ± 0.026^*^
F3	1.038 ± 0.062	32.139 ± 12.28	17.06 ± 1.637	0.826 ± 0.088
F3D	0.872 ± 0.169	29.405 ± 1.876	10.89 ± 3.782	0.824 ± 0.062

^*^Significant statistical difference compared to the respective control (*P *< 0.05).

**Table 4:** Consistency index (*K*) and flow index (*η*) of FLU-unloaded MEs (F1, F2, and F3) and FLU-loaded MEs (F1D, F2D, and F3D). Each value represents the mean (± standard deviation) of three replicates.

Formulations	*k* (Pa·s)	*η*
F1	1.14 ± 0.01	0.718 ± 0.003
F2	1.15 ± 0.04	0.840 ± 0.009
F3	8.44 ± 0.24	0.308 ± 0.007
F1D	1.39 ± 0.02	0.704 ± 0.004
F2D	2.73 ± 0.23	0.673 ± 0.020
F3D	6.27 ± 0.18	0.313 ± 0.007

**Table 5:** IC_30_ values against parasites for fluconazole samples and F1, F2, and F3 formulations with and without fluconazole.

Samples	IC_30_ (μM)
FLU	—
F1	—
F1D	55.62 ± 5.79
F2	—
F2D	42.19 ± 3.12
F3	—
F3D	30.00 ± 2.83^*^

^*^Significant statistical difference compared to the F1D and F2D formulations.
